# Heavy metals as endocrine disruptors

**DOI:** 10.55730/1300-0144.6132

**Published:** 2025-10-08

**Authors:** Arzu OR KOCA

**Affiliations:** Division of Endocrinology and Metabolism, Department of Internal Medicine, Dr. Abdurrahman Yurtaslan Ankara Oncology Education and Research Hospital, University of Health Sciences, Ankara, Turkiye

**Keywords:** Endocrine-disrupting agents, heavy metals, oxidative stress, metabolic disorders, endocrine diseases

## Abstract

Heavy metals are widely recognized as endocrine-disrupting agents primarily originating from anthropogenic sources and persisting in soil, air, and water. This review aims to present an updated and comprehensive synthesis of current evidence regarding the endocrine-disrupting effects of heavy metals, with particular emphasis on their roles in the pathogenesis of common endocrine and metabolic disorders. The toxicological impact of these elements depends on the level and duration of exposure, their chemical form, and the biological susceptibility of target tissues. Chronic exposure, even at low doses, has been implicated in the development of diabetes mellitus, obesity, autoimmune thyroiditis, thyroid cancer, pubertal disorders, and infertility through mechanisms involving oxidative stress, inflammation, receptor interference, and hormonal dysregulation. Epidemiological data and experimental studies consistently demonstrate that metals such as cadmium, lead, mercury, arsenic, and nickel can disrupt hormonal signaling and alter metabolic and reproductive homeostasis. Despite accumulating evidence, safe exposure thresholds remain undefined, and the long-term consequences of chronic low-dose exposure are still poorly understood. Understanding these mechanisms is crucial for developing preventive strategies and informing public health policies aimed at reducing the endocrine and metabolic disease burden associated with heavy metal exposure.

## 1. Introduction

Metals with a density typically greater than 5 g/cm^3^ are classified as heavy metals. Exposure to heavy metals, which represent a major subgroup of endocrine disruptors, has increased markedly over the past five decades and continues to rise in parallel with ongoing industrialization. Toxic effects may develop depending on the amount and duration of exposure to heavy metals such as chromium (Cr), cadmium (Cd), nickel (Ni), copper (Cu), zinc (Zn), lead (Pb), mercury (Hg), arsenic (As), manganese (Mn), cobalt (Co), and silver (Ag) [1]. Although several international organizations have established safety standards for exposure to these metals, adverse effects related to heavy metal toxicity are still frequently observed [2]. Mercury, arsenic, lead, chromium, aluminum, and cadmium are among the most common heavy metals responsible for toxicity in humans [1,3].

Some metals, such as copper, cobalt, iron, and zinc, are essential for various metabolic processes and biological signaling pathways. However, heavy metals such as arsenic, cadmium, lead, mercury, and nickel have no biological role in living cells, can be toxic even at very low concentrations, and are often classified as carcinogenic. Moreover, essential metals may also exert toxic and carcinogenic effects when present in excessive amounts [4].

The presence of heavy metals in ecosystems is primarily attributed to anthropogenic activities. Heavy metals, which naturally occur in the Earth’s crust as ores, are released during mining activities and subsequently dispersed into the environment. These metals persist in the soil and can be transported to other locations by air or water. Furthermore, certain heavy metals used in industrial processes can be released into the air through combustion or into the soil via wastewater. Industrial products such as cosmetics, paints, pesticides, and herbicides are also major sources of heavy metals [5]. The toxic effects of heavy metals may occur acutely or chronically following exposure through water, air, or food. The endocrine-disrupting effects of heavy metals are generally observed following chronic exposure, which may begin during the intrauterine period or early childhood. However, occupational exposure during adulthood or intense heavy metal exposure over a shorter period can also lead to endocrine-disrupting effects. Owing to the accumulation of heavy metals in biological tissues, toxic effects can be observed in various organs and systems (Table). This study specifically focuses on the endocrine system-disrupting effects of common heavy metals.

## 2. Effects of heavy metals on endocrine and metabolic disorders

### 2.1. Diabetes mellitus

The detection of elevated concentrations of various heavy metals in individuals with diabetes compared to nondiabetic individuals has led to the hypothesis that heavy metals may contribute to the development of diabetes [6]. However, it should be noted that the effects of most heavy metals on the pancreas have been investigated primarily through animal studies, experimental models, and a limited number of clinical trials.

Oxidative stress is known to contribute to pancreatic islet β-cell destruction and/or dysfunction in diabetes. Arsenic, historically described as “the poison of kings and the king of poisons,” is well known to induce oxidative stress [7]. In vivo and in vitro studies have demonstrated that arsenic plays a significant role in oxidative stress-induced pancreatic β-cell damage [8,9]. In mammals, chronic arsenic exposure decreases insulin secretion by impairing pancreatic β-cell function and is therefore associated with the development of diabetes [10]. Numerous studies have also shown that arsenic may alter signaling pathways involving nuclear factor kappa B (NFκB), p38 mitogen-activated protein kinase (MAPK), tumor necrosis factor-α (TNFα), phosphatidylinositol-3-kinase (PI3K) phosphorylation, and PI3K-dependent protein kinase B (PKB/Akt), thereby affecting insulin-stimulated glucose uptake in adipocytes and skeletal muscle cells, which may be associated with insulin resistance [11–13]. In β-cells exposed to high doses of arsenic, PI3K-mediated PKB/Akt phosphorylation—and consequently the activation of glucose transporter 4 (GLUT4) by insulin—are inhibited. It has been suggested that arsenic may mimic the effects of insulin through in vitro phosphorylation; hence, the role of arsenic in diabetes development has been extensively investigated [14].

Cadmium is a heavy metal well known to cause bone damage, referred to as Itai-itai disease, which leads to calcium loss and osteoporosis due to renal proximal tubule involvement and calcium malabsorption. Owing to the limited research assessing cadmium-associated decreases in insulin and increases in blood glucose levels, the relationship and underlying mechanisms of cadmium-induced diabetes remain unclear. Cadmium-induced oxidative damage is thought to contribute to diabetic symptoms through multiple mechanisms involving pancreatic β-cell dysfunction, altered insulin receptor activity, and lipid peroxidation [14]. Recent metaanalyses evaluating the association between cadmium exposure and glucose metabolism disorders have reported that elevated cadmium levels are significantly associated with an increased risk of diabetes and prediabetes. These analyses demonstrated that high cadmium exposure may increase the risk of diabetes by approximately 20%–30%, with comparable associations observed for both blood and urinary cadmium concentrations [15,16].

Chronic lead exposure has been implicated in the disruption of glucose homeostasis and in an increased risk of type 2 diabetes through mechanisms involving oxidative stress, systemic inflammation, and altered hepatic gluconeogenesis. Experimental studies have demonstrated that lead acetate exposure elevates hepatic expression of gluconeogenic enzymes such as phosphoenolpyruvate carboxykinase (PEPCK) and glucose-6-phosphatase (G6Pase), leading to fasting hyperglycemia and impaired glucose tolerance. Epidemiological data further suggest that higher blood lead concentrations are associated with an elevated prevalence of type 2 diabetes, although a direct causal relationship remains to be fully established. Variability across studies likely reflects differences in exposure dose, duration, and adjustment for confounding factors [17–19].

Several studies have shown that mercury, which is commonly encountered in daily life—from vaccine preservatives to fish—tends to occur at higher concentrations in individuals with diabetes than in nondiabetic individuals [20,21]. However, metaanalyses have not supported a causal relationship between mercury exposure and diabetes [22,23]. As with most heavy metals, the association between mercury and diabetes is thought to result from increased lipid peroxidation and oxidative stress [24]. Increased urinary glucose excretion has been reported in Minamata disease, which is characterized by toxic mercury exposure. This was associated with an increased prevalence of diabetes in Minamata disease [25,26]. However, high and toxic mercury levels are less common today than in the 1990s, and chronic low-dose exposure has also become relatively rare. Owing to numerous confounding factors in diabetes development, the mercury–diabetes relationship appears to involve complex mechanisms that have yet to be fully elucidated.

As with many other heavy metals, nickel—often found in combination with elements such as sulfur, iron, and arsenic—has been reported to increase free radical levels and lipid peroxidation [27]. It has also been shown to increase hepatic glycolysis and pancreatic glucagon release in rats, decrease peripheral glucose utilization, and potentially induce gluconeogenesis [28]. Despite evidence of nickel-induced hyperglycemia, a study reporting that nickel may prevent streptozotocin- and alloxan-induced hyperglycemia suggests that the nickel–diabetes relationship remains controversial [29].

The zinc–diabetes relationship differs from that of other heavy metals, as zinc deficiency leads to decreased insulin levels and increased glucose tolerance, whereas other heavy metals show a direct causal association with diabetes. Zinc supplementation has been shown to be effective in the prevention and treatment of type 1 and type 2 diabetes in rodents. Zinc and its associated metallothioneins have been shown to reduce oxidative stress-related diabetic complications. Furthermore, the zinc transporter protein zinc transporter 8 (ZnT8) has been suggested to play an essential role in both zinc accumulation and the regulation of insulin secretion in pancreatic β-cells. Genomic studies have identified associations between diabetes and zinc transporter–related genes [14]. However, the effects of excessive zinc intake on the pancreas remain unclear based on current evidence.

### 2.2. Obesity

Obesity represents a major public health crisis, as it is directly associated with numerous diseases and independently contributes to morbidity and mortality. It is also associated with numerous environmental and genetic factors. Given that most endocrine disruptors target adipose tissue as a primary site of accumulation, it is not surprising that endocrine-disrupting effects are more frequently observed in individuals with obesity, along with higher detected levels of heavy metals in these individuals. Epidemiological studies have demonstrated an association between heavy metal exposure and the prevalence of obesity [30]. The key question is whether individuals with obesity experience greater heavy metal–related toxic effects due to increased adipose tissue mass, or whether excessive heavy metal exposure contributes to the development and progression of obesity. Findings from animal studies indicate that heavy metals predispose to obesity by inducing endoplasmic reticulum stress, disrupting the hypothalamic dopaminergic system, and promoting inflammation [31–33].

Differences in genetic background and levels of environmental chemical pollution across populations may account for the variability in the types and concentrations of heavy metals associated with obesity. For instance, seafood is one of the primary sources of mercury and total arsenic intake in populations that consume large amounts of fish and shellfish. This may influence the etiology of obesity. However, cadmium exposure tends to be higher in populations that frequently consume vegetables, cereals, fruits, and dairy products [34].

Experimental in vivo studies have demonstrated significant effects of mercury, cadmium, lead, and arsenic exposure on adipose tissue growth. However, in vitro studies have revealed that these heavy metals can both promote and inhibit adipogenesis through abnormal regulation of key adipogenic pathways. In a review by Tinkov et al. (2021), who have conducted numerous studies on the effects of heavy metals, this phenomenon was explained as follows: “Low-dose heavy metal exposure primarily promotes an increase in adipocytes, whereas high-dose exposure inhibits adipocyte differentiation” [30]. According to a study by Sun et al., dose-dependent weight gain was observed in rats exposed to lead for only 3 weeks [35]. The observations that (1) even a single heavy metal can affect weight gain and (2) multiple heavy metals promote ectopic adipose tissue accumulation by inhibiting adipocyte differentiation suggest that the involvement of heavy metals and other endocrine disruptors in the global rise of obesity may be more significant than previously recognized.

### 2.3. Thyroid disorders

The incidence and prevalence of thyroid cancer are also increasing worldwide. The primary reason for this increase is likely related to overdiagnosis. This increase may also be partially attributed to exposure to solvents, plastic compounds, and heavy metals associated with modern industrial activities [4]. One of the most striking observations related to heavy metals is the increased incidence of thyroid cancer in the volcanic region surrounding Mount Etna in Sicily [36]. It has been reported that the twofold higher incidence of thyroid cancer in volcanic regions compared to nonvolcanic areas is due to higher concentrations of heavy metals in water, air, and plants [37]. Lifelong chronic exposure to heavy metals among individuals living in volcanic regions has been associated with an increased incidence of thyroid cancer, particularly the papillary subtype [38]. This effect has been attributed to three potential mechanisms: (1) thyroid follicular cells operate in a constant oxidative environment due to their dependence on hydrogen peroxide, resulting in continuous oxidative stress exposure; (2) thyroid cells have a slow turnover rate, allowing mutations to accumulate; and (3) thyroid cells are inherently more susceptible to damage by exogenous toxicants. It should be emphasized that immature thyrocytes in the maturation stage are more susceptible to heavy metal exposure than mature thyrocytes [4].

Another important effect of heavy metals on the thyroid gland is their potential to induce thyroid autoimmunity. Observational epidemiological studies have shown a higher prevalence of autoimmune thyroid disease, particularly among individuals exposed to aluminum, mercury, nickel, and cadmium compared to those without such exposure [39–42]. The effects of heavy metals on thyroid function are attributed to their capacity to induce thyrocyte dysfunction and are frequently associated with elevated thyroid-stimulating hormone (TSH) levels and reduced thyroxine concentrations, although the underlying mechanisms remain unclear [43,44]. It has been suggested that measured serum thyroid hormone levels may also be associated with decreased thyroid-binding protein concentrations [45].

### 2.4. Gonadal and reproductive disorders

As with many endocrine disruptors, heavy metals are among the first toxic agents recognized to influence gonadal function. Studies have shown that heavy metals disrupt gonadal function and the reproductive cycle and can also induce physiological stress [38,46,47]. Gonads are affected by heavy metals differently from other organs, and it has been suggested that they may act as a biological barometer of toxic exposure. The release of cadmium, lead, and mercury into the environment following various anthropogenic activities has been linked with infertility [48].

Arsenic has been shown to impair sperm motility and reduce semen volume [49]. Pregnant rats exposed to arsenic have been reported to exhibit increased hypothalamic levels of gonadotropin-releasing hormone (GnRH), with their offspring showing a tendency toward early puberty [50]. In addition, high environmental arsenic exposure in pregnant women has been associated with increased rates of spontaneous abortion and stillbirth [51].

Elevated blood mercury levels in girls may trigger early menarche [52]. Premature puberty has been observed in children of mothers exposed to high levels of mercury during the prenatal period [53]. Literature reviews suggest that mercury exposure may be associated with adverse pregnancy outcomes in women, depending on the dose and duration of prenatal exposure. In men, has been shown to affect semen quality—reducing sperm count, motility, and altering morphology—which may lower fertility potential [54].

Elevated blood lead levels have been associated with decreased sperm count, impaired motility, and abnormal sperm morphology [55,56]. Furthermore, high levels of lead exposure can disrupt the hypothalamo-hypophyseal axis and have been reported to delay puberty and growth [57].

Cadmium is another heavy metal that may exert detrimental effects on gonadal function. Studies have reported that cadmium reduces sperm count, motility, and concentration, and decreases testicular volume. In some fish species, a negative correlation has been observed between high cadmium levels and sperm count, density, and semen volume in the ejaculate [58,59]. In male children, elevated cadmium levels have been associated with delayed puberty, reduced serum testosterone levels, and decreased testicular volume [60]. However, several studies examining human populations have found no significant evidence of adverse effects associated with cadmium exposure [48,61].

## 3. Conclusion

Despite the vast array of complex chemical mixtures to which humans are exposed in this era of rapid industrialization and escalating chemical pollution, most studies on endocrine disruptors focus on the effects of individual compounds. Moreover, most of the available evidence is derived from cell culture and animal studies. Current evidence on the endocrine-disrupting effects of heavy metals is limited by methodological challenges and inconsistencies. There are no established standards for safe levels of most heavy metals, and it remains difficult to assess the impact of chronic, low-dose exposure to these pollutants on the endocrine system. Heavy metals may contribute to the pathogenesis of several highly prevalent endocrine diseases, including diabetes mellitus, obesity, autoimmune thyroiditis, thyroid cancer, and infertility; however, the extent of these effects remain unclear.

## Figures and Tables

**Table:** Heavy metals, associated endocrine and metabolic disorders, and their mechanisms of action described in the current review.

Heavy metal	Associated endocrine and metabolic disorders	Mechanisms of action
**Arsenic (As)**	Diabetes mellitus, thyroid dysfunction, infertility, and early puberty	Induces oxidative stress; damages pancreatic β-cells and reduces insulin secretion; alters NFκB, MAPK, TNFα, and PI3K/Akt signaling pathways; impairs GLUT4 activation; and affects thyroid hormone synthesis as well as hypothalamic GnRH regulation.
**Cadmium (Cd)**	Diabetes mellitus, obesity, thyroid autoimmunity, and hypogonadism	Induces oxidative stress and lipid peroxidation; impairs pancreatic β-cell and insulin receptor function; disrupts lipid metabolism; and is associated with thyroid autoimmunity and reduced reproductive hormone levels.
**Lead (Pb)**	Type 2 diabetes, obesity, delayed puberty, and infertility	Induces oxidative stress and inflammation; upregulates hepatic gluconeogenic enzymes (PEPCK and G6Pase); alters glucose metabolism; and disrupts hypothalamo-pituitary-gonadal axis, leading to delayed puberty and impaired fertility.
**Mercury (Hg)**	Diabetes mellitus, thyroid autoimmunity, early puberty, and adverse pregnancy outcomes	Induces lipid peroxidation and oxidative stress; alters thyroid-binding proteins and thyroid hormone levels; is associated with premature puberty and impaired semen quality; and may contribute to fetal loss at high exposure levels.
**Nickel (Ni)**	Diabetes mellitus and thyroid autoimmunity	Increases free radical generation and lipid peroxidation; impairs glucose utilization and insulin signaling; and is associated with immune-mediated thyroid dysfunction.
**Zinc (Zn)**	Diabetes mellitus (protective effect)	Zinc deficiency decreases insulin levels and increases glucose intolerance; zinc supplementation enhances β-cell function and reduces oxidative stress; and the ZnT8 transporter gene is linked to insulin regulation.
**Chromium (Cr)**	Thyroid cancer	Associated with thyroid carcinogenesis related to environmental exposure; may exert oxidative and mutagenic effects in thyroid follicular cells.
**Copper (Cu)**	Essential trace element; toxic in excess	Excessive levels cause oxidative stress and may disrupt thyroid hormone synthesis (essential trace element but toxic when accumulated).
**Manganese (Mn)**	Not directly linked to any endocrine or metabolic disorder in the text	Mentioned among common heavy metals associated with human toxicity; no specific endocrine mechanism discussed in the text.
**Cobalt (Co)**	Not specifically discussed	Listed among common heavy metals with potential endocrine toxicity; specific mechanisms were not detailed in the manuscript.

## Abbreviations

PKB/Akt: protein kinase B
GnRH: gonadotropin-releasing hormone
MAPK: mitogen-activated protein kinase
NFκB: nuclear factor kappa B
PEPCK: phosphoenolpyruvate carboxykinase
PI3K: phosphatidylinositol-3-kinase
ROS: reactive oxygen species
TSH: thyroid-stimulating hormone
